# Socioeconomic differentials of neonatal mortality in Northern Karnataka: implications

**DOI:** 10.1186/1753-6561-6-S5-P6

**Published:** 2012-09-28

**Authors:** Jyothi Hallad, Arin Kar, Jayanna Krishnamurthy, Prem Mony, BM Ramesh

**Affiliations:** 1 Population Research Centre, JSS Institute of Economic Research, Dharwad, Karnataka, India; 2 Karnataka Health Promotion Trust, Bangalore, India; 3 St. Johns National Academy of Health Sciences, Bangalore, India

## Introduction

Reduction of neonatal mortality from the current levels of 29 neonatal deaths per 1000 live births to 21 per 1000 live births is one of the major goals of the National Rural Health Mission (NRHM) in Karnataka. Strategic focus on geographies and sub-populations within the state where neonatal mortality is the highest will enhance the speed and magnitude of its reduction. A recently completed household survey in the eight districts of north Karnataka (Bagalkot, Bellary, Bidar, Bijapur, Gulbarga, Koppal, Raichur and Yadgir) conducted by project *Sukshema* provides an opportunity to examine the sub-population differentials in neonatal mortality. The *Sukshema* project supports the government of Karnataka through the development and adoption of effective operational and health system approaches within the NRHM.

## Methods

We conducted a survey using two-stage systematic stratified sampling. In the first stage, 167 villages were sampled using probability proportion to population size method. In the second stage, 30 households with currently married woman age 15-34 years were selected systematically with equal probability and without replacement - an equal number of scheduled caste/tribe (SC/ST) population and non-SC/ST households, based on the household listing in the selected villages. During the household interviews, the respondents were asked for details of deaths in the household since January 2009. A total of 4972 households were interviewed with a response rate of 99%. The infant and neonatal mortality rates were computed separately for the SC/ST and non-SC/ST samples based on the births since January 2009.

## Results

While the estimated infant mortality rates are somewhat similar in the two groups (62 and 58 per 1000 live births among the SC/ST and non-SC/ST groups respectively), the neonatal mortality is substantially higher among the SC/ST group (49 compared to 39 per 1000 live births). Preliminary analyses indicates that most of these differences in neonatal mortality are due to the differential nutritional status during pregnancy, differential rates in home deliveries, differential quality of care during delivery and immediate postpartum period due to the choice of facilities for delivery (public vs*.* private).

## Discussion

Caste differentials in neonatal mortality strongly indicate the need to focus on the nutritional status of SC/ST women in general and during pregnancy in particular, facilitating institutional deliveries and improving the quality of care during delivery and immediate postpartum period in public facilities.

## Competing interests

None declared

## Funding statement

The study was funded by the Bill and Melinda Gates Foundation.

